# SBS Modified Bitumen with Organic Layered Double Hydroxides: Compatibility and Aging Effects on Rheological Properties

**DOI:** 10.3390/ma14154201

**Published:** 2021-07-27

**Authors:** Canlin Zhang, Hongjun Dong, Zhengli Yan, Meng Yu, Ting Wang, Shi Xu, Zhenliang Jiang, Changbin Hu

**Affiliations:** 1 College of Civil Engineering, Fuzhou University, Fuzhou 350108, China; zhangcl@fzu.edu.cn (C.Z.); 200520118@fzu.edu.cn (H.D.); 051901527@fzu.edu.cn (Z.Y.); huchangbin@fzu.edu.cn (C.H.); 2 School of Advanced Manufacturing, Fuzhou University, Fuzhou 350108, China; 3 State Key Laboratory of Special Functional Waterproof Materials, Beijing Oriental Yuhong Waterproof Technology Co. Ltd., Beijing 101309, China; yumeng@yuhong.com.cn; 4 Testing Center of Fuzhou University, Fuzhou 350108, China; wting@fzu.edu.cn; 5 Civil Engineering and Geosciences, Delft University of Technology, 2628 CN Delft, The Netherlands; 6 Department of Civil and Environmental Engineering, The Hong Kong University of Science & Technology, Hong Kong 999077, China

**Keywords:** SBS modified bitumen, layered double hydroxides, organic modification, rheological properties, aging resistance

## Abstract

SBS-modified bitumen (SMB) is susceptible to aging, which seriously influences its service performance and life. In order to strengthen the anti-aging ability of SMB, triethoxyvinylsilane was designed to organically modify layered double hydroxides (LDHs) and was applied to modify SMB. The dispersibility and storage stability of LDHs in SMB were markedly enhanced after triethoxyvinylsilane organic modification, and the compatibility and storage stability of SBS in bitumen were simultaneously enhanced. Compared with SMB, the introduction of LDHs and organic LDHs (OLDHs) could ameliorate the high-temperature properties of SMB, and the thermostability of SBS in bitumen at a high temperature was also distinctly improved, especially OLDHs. After aging, due to the oxidation of molecular bitumen and the degradation of molecular SBS, SMB became hardened and brittle, and the rheological properties were significantly deteriorated, which had serious impacts on the performance of SMB. LDHs can mitigate the detriment of aging to bitumen and SBS, and the deterioration of the rheological properties of SMB is obviously alleviated. As a result of the better dispersibility and storage stability, OLDHs exerted superior reinforcement of the anti-aging ability of SMB.

## 1. Introduction

The unsatisfactory high-temperature and low-temperature performance of base bitumen have promoted the extensive application of styrene-butadiene-styrene (SBS) as a bitumen modifier in highway pavement [1,2,3]. However, due to the exposure to heat, oxygen and ultraviolet (UV) light throughout its service life, SBS-modified bitumen (SMB) will suffer thermal-oxygen aging and UV aging, which causes damage to bitumen’s properties, such as cracking and stripping, thereby degrading the service performance and life of bituminous pavement [4,5,6,7,8,9,10]. Hence, it is of considerable necessity to acquire SMB with outstanding aging resistance.

Some studies have engaged in alleviating the aging of SMB, and the most widespread method is using additives [11,12]. Zhang et al. [13] found that a large dosage of sodium-montmorillonite (MMT) can enhance the aging resistance of SMB. However, the low-temperature performance of SMB was decreased after adding a large amount of MMT. Feng et al. [14] discovered that UV absorbers can reduce the UV aging of SMB, but the effect of UV absorbers was not universal, which only worked for specific types of SMB. In addition, some inorganic nanomaterials were reported to improve the aging resistance of SMB in some research, such as nano-SiO_2_, nano-ZnO, nano-TiO_2_ and so on [15,16]. However, the dispersity of inorganic nanomaterials in SMB was poor, which limited their application in SMB.

**Figure 1:**
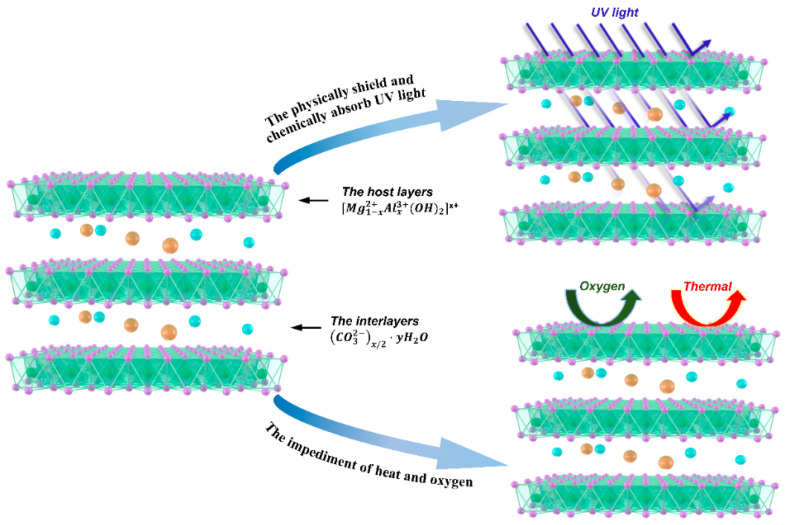
The mechanism of LDHs in improving the UV aging resistance and thermo-oxidative aging resistance.

Layered double hydroxides (LDHs) are a kind of supramolecular structural material. Compared with other modifying agents, LDHs can not only physically shield and chemically absorb UV light, but also can impede the penetration of heat and oxygen into bitumen, because of its unique structure [17,18,19]; this mechanism is shown in Figure 1. Hence, in recent years, LDHs have been used as an anti-aging modifier and applied in bitumen modification. Wu et al. [20] proved that LDHs can alleviate the damaging effects of UV light on the rheological properties, and strengthen the anti-UV aging properties of bitumen. Xu et al. [21] found that LDHs can reduce bitumen aging and reduce SBS degradation. However, as an inorganic material, the strong hydrophilicity of LDHs can result in poor storage stability in bitumen [22]. Additionally, large amounts of superficial hydroxyl groups in LDHs would promote the agglomeration among LDHs and the separation between LDHs and bitumen [23]. These limitations can remarkably restrict the popularization of LDHs in resisting aging of bitumen. Silane coupling agent applied to surface-modified LDHs, using a surface organification method, was found to change the surface of LDHs from hydrophilic to hydrophobic, providing LDHs with better storage stability in fresh bitumen [24,25]. Therefore, the surface organic modified method might enhance the storage stability of LDHs and improve the anti-aging performance in SMB.

In this paper, silane coupling agent was utilized to organically modify to surface of LDHs, and was applied in SMB modification. The compatibility and storage stability of organic LDHs in SMB were evaluated, and the effect of LDHs and OLDHs on the rheological properties of SMB before and after aging were thoroughly investigated.

## 2. Materials and Methodologies

### 2.1. Materials

Virgin bitumen (AH 70) was acquired from Fuzhou Development Zone Lugang Asphalt Company Limited (Fuzhou, China), SBS was captured from the Baling branch of Sinopec Company Limited (Yueyang, China), and their basic properties are listed in Table 1. LDHs were purchased from Ruifa Chemical Company Limited (Jiangyin, China). Triethoxyvinylsilane was produced by Shandong Huanzheng Chemical Company Limited (Jinan, China).

### 2.2. Sample Preparation

The LDHs and triethoxyvinylsilane were dispersed in alcoholic aqueous solution separately. Then, these two solutions were mixed successively at 50 °C for 3 h and 70 °C for 0.5 h. Afterwards, the white LDH slurry was repeatedly washed by vacuum filtration, and then the filter residue was dried at 80 °C for 12 h. Ultimately, the dried product was crushed and ground into powdered materials at a sieve size of 200 mesh.

The modified bitumen samples were prepared using the melt blending method. Firstly, virgin bitumen was heated to fluid at 180 °C. Then, 1–5% (weight of bitumen)LDHs powders and 4% (weight of bitumen) SBS were dumped into bitumen samples, respectively, and the mixtures were subsequently sheared at the shearing speed of 4000 rpm and 180 °C for 60 min (CY-028, Chengyi-Machinery Co., Wenzhou, China). Finally, the mixtures were transferred to the low-speed mechanical agitation for 90 min.

### 2.3. Aging Procedures

The aging of all binder samples was simulated by a thin film oven test (TFOT) and UV irradiation, successively. TFOT was implemented according to ASTM D 1754. The remnant samples from TFOT continued to be radiated by UV lights at the intensity of 2500 μW/cm^2^ at 60 °C for 7 days in an UV lamp oven.

### 2.4. Storage Stability Test

The storage stability of LDHs and SBS in bitumen is critical for the properties of SMB. The storage stability test was conducted following ASTM D 5976. Noteworthily, SBS will swell and float in bitumen, which results in the softening point of the top section increasing in the storage stability test. On the contrary, LDHs will aggregate and sedimentate in bitumen, which causes the softening point increase of the bottom section. The traditional evaluation criterion is the softening point difference value of the top and bottom sections (ΔS) in the storage stability test; the smaller the ΔS, the better the storage stability. Due to the opposite dissociation of LDHs and SBS in bitumen, the traditional evaluation criterion is not appropriate for the SMB containing LDHs (LSMB) and OLDHs (OLSMB)—the smaller ΔS did not mean that LSMB or OLSMB possessed better storage stability. Hence, the evaluation criterion was amended in this paper; the softening points of all three sections were tested (*S_top_*, *S_middle_*, *S_bottom_*), and the computing method of ΔS of SMB, LSMB and OLSMB in this paper was as follows:

ΔS = |*S_top_* − *S_middle_*| + |*S_bottom_* − *S_middle_*| (1)

### 2.5. Fluorescence Microscope Test

The fluorescence microscope (BXF-150, Bingyu Co., Shanghai, China) was used to observe the morphology of SMB, LSMB, and OLSMB. Firstly, a drop of fluid bitumen sample was placed on the glass slide, and then a cover glass was superposed upon it, forming a homogeneous bitumen film. Finally, the polymer (i.e., SBS) dispersion state in the bitumen film was observed by the fluorescence microscope. Generally, yellow-green fluorescence represents the SBS phase and black represents the other phases (e.g., bitumen and non-polymers) [26,27].

### 2.6. Rheological Properties Test

The rheological properties (storage modulus, loss modulus, phase angle, fatigue factor) of SMB, LSMB, and OLSMB samples at different temperature ranges and frequencies were measured by DSR (Dynamic Shear Rheometer, MCR101, Anton Paar Co., Graz, Austria). The temperature sweep and shear creep modes were utilized, and the main parameters are shown in Table 2.

The creep stiffness (S) and creep rate (m-value) of bitumen samples were evaluated by the BBR test (Bending Beam Rheometer, Canton, TE-BBR, PA, USA). The binder beam was placed on two holders with three-point bending, and the test was conducted at different temperatures (−12, −18, and −24 °C) with a loading time of 60 s.

## 3. Results and Discussion

### 3.1. Compatibility and Storage Stability

**Figure 2:**
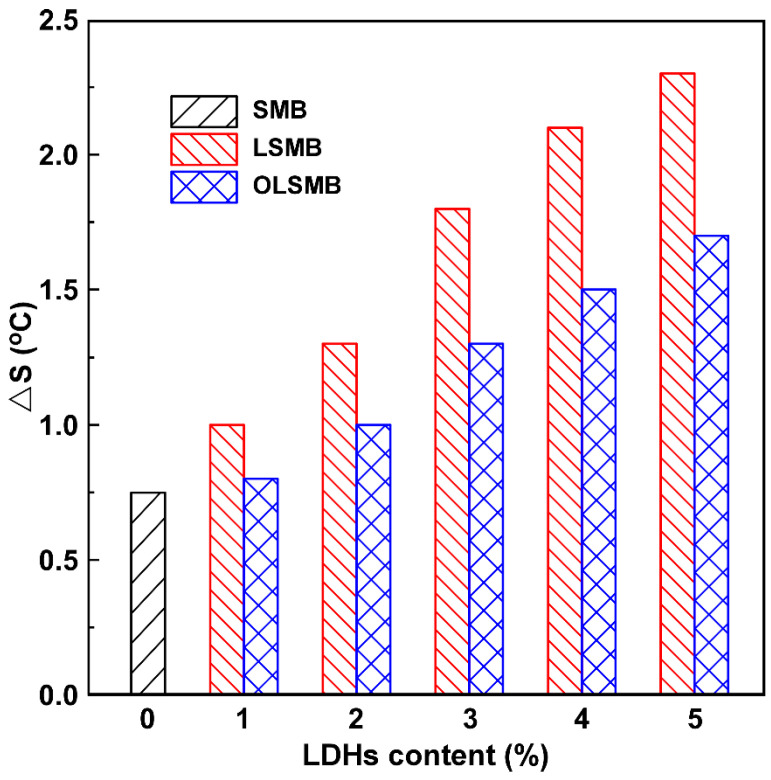
The storage stability of SMB, LSMB and OLSMB.

The storage stability of LDHs in SBS-modified bitumen plays a key role in the rheological and anti-aging properties of modified bitumen. Hence, LSMB or OLSMB should possess better storage stability. The storage stability of SMB, LSMB, and OLSMB is illustrated in Figure 2. In comparison with SMB, it can be observed that the ΔS of LSMB or OLSMB increased, and the tendency became more noticeable with the increasing dosage of LDHs or OLDHs, which was due to the sinking of LDHs in bitumen during storage, resulting in the softening point increase of the bottom. Furthermore, compared with LSMB, the ΔS of OLSMB was much smaller, and it exhibited a more remarkable value at a large dosage of LDHs, which indicated that OLSMB had better storage stability than LSMB. In our previous research, it was found that organic modification with silane coupling agent has two benefits for LDHs. On the one hand, the surface hydrophilic groups (hydroxide radical) of LDHs were reduced after silane coupling agent organic modification, which can inhibit the aggregation between LDH particles and improve the dispersiveness in SMB. On the other hand, some organic groups in silane coupling agent have been introduced into LDHs, which can increase the lipophilicity of LDHs and enhance the storage stability of LDHs in SMB [24,25,28]. Because of these two aspects, the dispersity and storage stability of LDHs in SMB have been significantly enhanced after triethoxyvinylsilane organic modification.

**Figure 3:**
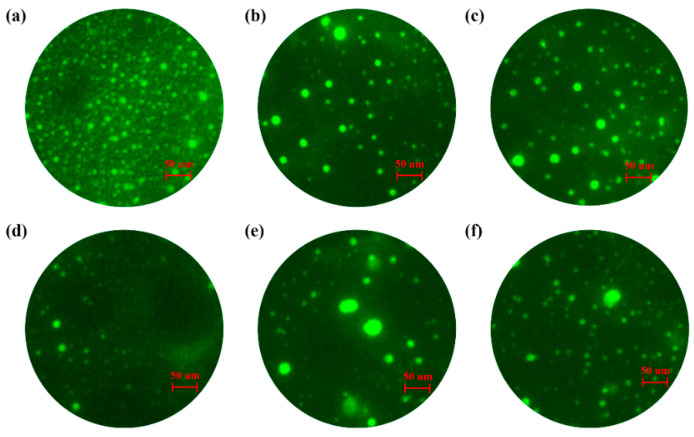
The fluorescence microscope images of the top parts (**a**–**c**) and bottom parts (**d**–**f**) of SMB, LSMB and OLSMB.

The fluorescence microscope photographs of SMB, LSMB, and OLSMB samples after the storage stability test are shown in Figure 3. It is widely accepted that the fluorescence of SMB is due to the SBS, and the more intense the fluorescence, the higher the content of SBS in bitumen. The fluorescence photographs of SMB (the top section (Figure 3a) and the bottom section (Figure 3d) are significantly different—the fluorescence of the top section (Figure 3a) was more intense than that of the bottom section (Figure 3d), which was due to the swelling and floating of SBS in bitumen. Compared with SMB, the fluorescence difference of LSMB and OLSMB (Figure 3b,e for LSMB, Figure 3c,f for OLSMB) was distinctly weakened, especially OLSMB. As an inorganic material dispersing in SMB, LDHs will impede the movement of SBS in bitumen to a certain extent, which is beneficial to restrain the floating of SBS in bitumen, and improve the storage stability of SBS in bitumen. As a result of the better dispersity and storage stability of OLDHs in SMB, the interaction between LDHs and SBS was strengthened, and hence, the distribution of SBS in OLSMB became more uniform and stable.

### 3.2. Rheological Properties

#### 3.2.1. Low-Temperature Sweep

**Figure 4:**
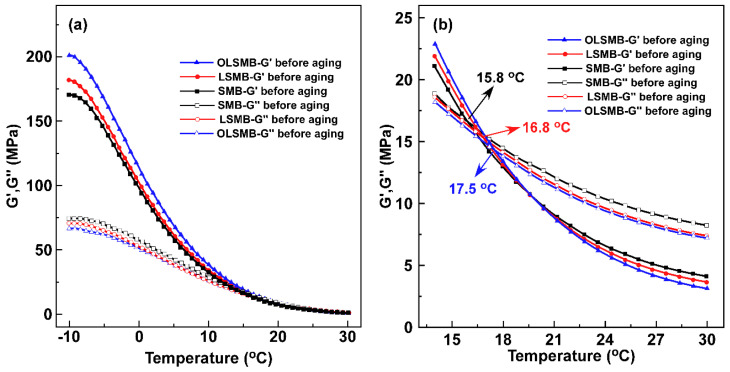
G′ and G″ vs. temperature from (**a**) −10 to 30 °C and (**b**) 14 to 30 °C and the cross points for SMB, LSMB and OLSMB before aging.

The curves of storage modulus (G′) and loss modulus (G″) from −10 to 30 °C of all binder samples before aging are displayed in Figure 4. In Figure 4a, it can be observed that both the G′ and G″ decreased incrementally with the increase in temperature. For all binder samples, the G′ was obviously larger than the G″ in the beginning; however, due to the larger decreasing trend of G′ than that of G″ with the increase in temperature, the size order of G′ and G″ changed in the end; there was a cross point between G′ and G″, and the temperature corresponding to the cross point was the turning point of bitumen from more elastic behavior to more viscous behavior. Namely, bitumen samples exhibited more elastic behavior before the cross point; after that, more viscosity was shown. The temperatures corresponding to the cross points of all binder samples are depicted in Figure 4b; compared with SMB, the temperature of LSMB and OLSMB increased by varying degrees, which was due to the incorporation of LDHs or OLDHs, increasing the elastic behavior of bitumen.

**Figure 5:**
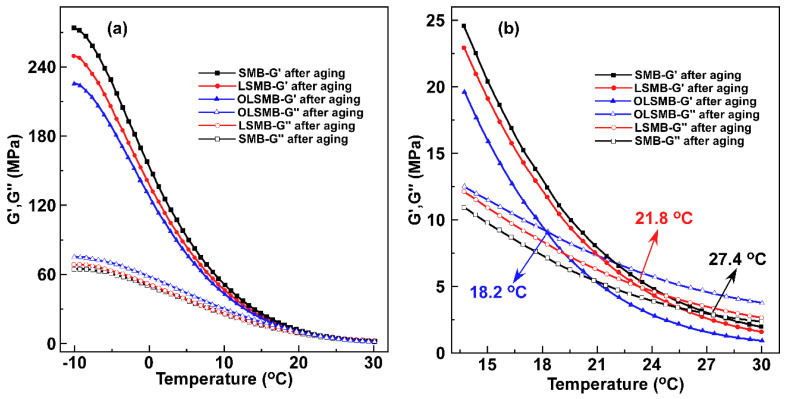
G′ and G″ vs. temperature from (**a**) −10 to 30 °C and (**b**) 14 to 30 °C and the cross points for SMB, LSMB and OLSMB after aging.

After aging, the curves of G′ and G″ for all binder specimens are presented in Figure 5. The G′ of all specimens increased by varying degrees, and the G″ correspondingly decreased. That is, the elastic behavior of bitumen samples notably increased after aging, and the viscous behavior decreased [29]. In contrast with the G′ and G″ before and after aging, it can be found that the variation of SMB was the most significant, followed by LSMB, and OLSMB had the lowest variation. Furthermore, the G′ and G″ variation before and after aging resulted in the temperature corresponding to the cross point, demonstrating a different change (as shown in Table 3); when the temperature of SMB increased from 15.8 to 27.4 °C (the temperature increment of 11.6 °C), it remarkably delayed the conversion of SMB from more elastic behavior to more viscous behavior, which was unfavorable for the low-temperature performance of bitumen. Compared with SMB, the temperature increments of LSMB and OLSMB obviously decreased—those of the LSMB to 5.0 °C, and those of the OLSMB to 0.7 °C. The result indicate that LDHs and OLDHs can reduce the aging impact on the bituminous viscoelasticity at a low temperature, especially OLDHs.

#### 3.2.2. High-Temperature Sweep

**Figure 6:**
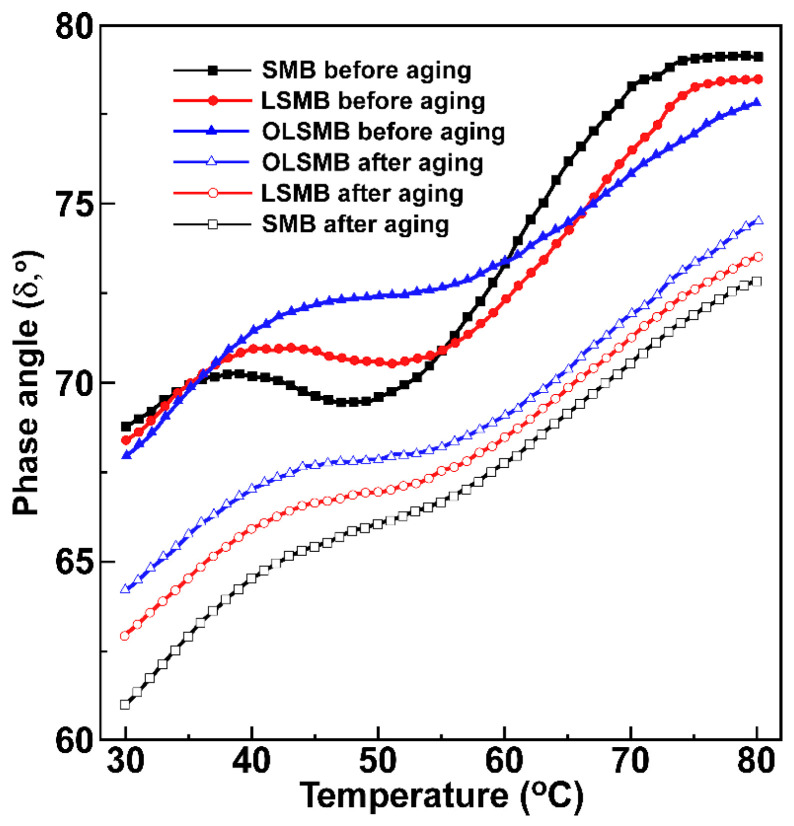
Phase angle of SMB, LSMB and OLSMB at a medium and high temperature before and after aging.

The phase angles (δ) of all bitumen samples at a medium and high temperature (from 30 to 80 °C) are displayed in Figure 6. With the increase in temperature, the δ of all samples increased. It is noteworthy that an δ plateau can be observed in SMB; that is, δ of SMB was almost unchanged and even decreased at the temperature ranging from 40 to 50 °C. The reason for this phenomenon is the network structure formation of SBS, resulting in the elastic behavior of SMB increasing. Compared with SMB, a similar δ plateau can be found in LSMB and OLSMB. The difference was that the δ change of LSMB became smooth in this area; moreover, the δ was higher than that of SMB, and this might be because the dispersion of LDHs in bitumen hindered the movement of SBS molecular, which caused the network structure formation temperature of SBS moving to a higher temperature. This change in OLSMB was more obvious than that in LSMB, which is due to the better storage stability of OLDHs in SMB. In addition, this area of OLSMB moved to the high-temperature area, which was conducive to strengthening the high temperature stability of SBS in SMB.

After aging, SMB hardened, and the δ of all bitumen samples decreased. The δ of SMB was the lowest after aging, LSMB was next, and that of OLSMB was the highest, which is contrary to that before aging. Furthermore, in the SMB and LSMB, the δ plateau before aging vanished after aging, because of the molecule degradation of SBS in the aging process. Noteworthily, the δ plateau can be found in the OLSMB after aging. This result indicates that OLDHs showed excellent improvement in the aging resistance of SMB, and they can concurrently alleviate the damage caused by aging in bitumen and the degradation caused by aging of SBS.

#### 3.2.3. Frequency Sweep Test

**Figure 7:**
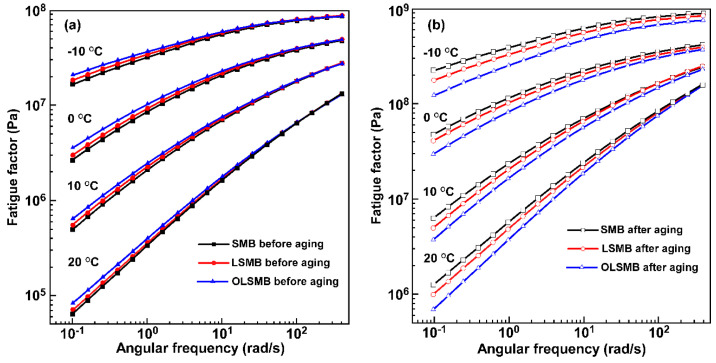
Fatigue factor vs. frequency for SMB, LSMB and OLSMB (**a**) before and (**b**) after aging.

The fatigue factor (G*·sin δ) is used to evaluate the fatigue resistance of SMB. The higher the G*·sin δ, the lower the fatigue resistance [30,31]. The frequency correlations of G*·sin δ at different temperatures (−10, 0, 10, and 20 °C) are demonstrated in Figure 7. It can be found that the G*·sin δ values of all modified binder samples increased with the decrease in temperature and the increase in frequency, which indicated that the modified binder tended to demonstrate fatigue cracking at a low-temperature and high loading frequency. In contrast with SMB, the G*·sin δ values of LSMB and OLSMB slightly increased. This indicates that LDHs were not conducive to the fatigue cracking of SMB, which was due to the increase in the elastic properties of the binder after the affiliation of LDHs. Fortunately, the effect was slight due to the small dosage.

After aging, the G*·sin δ of the three binders increased at different levels—that of SMB was the highest, and that of OLSMB was the lowest. The G*·sin δ ranked in the order of SMB > LSMB > OLSMB, and the lower the temperature and frequency, the more remarkable the discrepancy of the three binders. It was noteworthy that G*·sin δ of OLSMB shifted from the highest before aging to the lowest after aging, while the G*·sin δ of SMB demonstrated the opposite shift. These results imply that the aging resulted in the fatigue resistance deterioration of SMB, and the addition of OLDHs and LDHs can improve the fatigue resistance ability of SMB; moreover, OLDHs exhibited a more prominent effect than that of LDHs.

#### 3.2.4. Bending Beam Rheometer (BBR) Test

**Figure 8:**
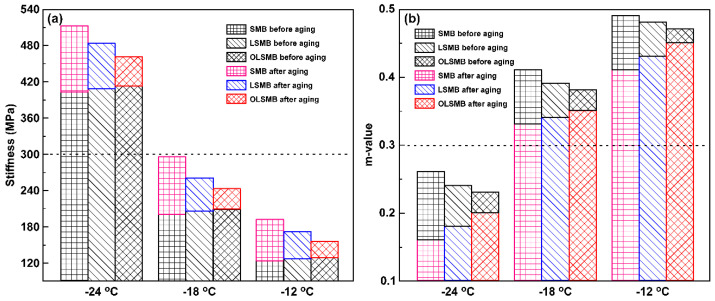
Creep stiffness (**a**) and m-value (**b**) of all binders before and after aging.

As previously analyzed, the aging will result in the hardening and brittleness of SMB. This phenomenon is detrimental for the low-temperature cracking of SMB, which causes the premature failure of SMB at low temperatures. To assess the low-temperature anti-cracking effects of all binders before and after aging, the creep stiffness (S) and creep rate (m-value) were utilized in this paper. S reflects the anti-low temperature deformation ability of binders, m-value represents the stress relaxation property of binder samples at low temperature. Generally, the higher the S and the smaller the m-value, the worse the anti-cracking of SMB at low temperatures [32,33]. The S and m-value of all binders before and after aging are illustrated in Figure 8. It can be found that as the S of all binders increased, the m-value correspondingly reduced with the decreasing temperature, which resulted in a relatively elevated risk of cracking at a comparatively low temperature. In comparison with SMB, the S of LSMB and OLSMB increased slightly; meanwhile, the m-value reduced marginally, because of LDHs and OLDHs serving as elastic constituents in SMB. The change in the S and m-value for LSMB and OLSMB was prejudicial to the low-temperature cracking; noteworthily, the influence was very limited.

After aging, the S of all binder samples increased visibly, and the m-value reduced simultaneously, and the lower the temperature, the more significant this was. This indicates that the risk of cracking for binders markedly increased, especially at lower temperatures. In addition, it can be clearly observed that the S and m-value of the three binders presented a diverse variation trend. SMB was the most obvious; the S moved from the lowest of the three before aging to the highest after aging, and the m-value moved from the highest before aging to the lowest after aging. This value was followed by that of LSMB. OLSMB was the lowest, and the S and m-value of OLSMB before and after aging showed the opposite variation with that of SMB. The results show that the incorporation of LDHs can prevent aging’s impact on the low-temperature cracking of SMB, and heighten the anti-aging capacity of SMB; the effectiveness of LDHs has been further promoted after triethoxyvinylsilane organic modification.

The service temperature limit of bitumen depends on the minimum temperature that bitumen satisfies the demand of m ≥ 0.3 and S ≤ 300 MPa [34,35]. As seen in Figure 8, all binders complied with a −18 °C limited temperature, but did not conform to a −24 °C limited temperature. This indicates that the service temperature limit of all three modified bitumens was not reduced, although OLSMB behaved with favorable low-temperature cracking resistance. To sum up, both LDHs and OLDHs can strengthen the anti-aging ability of SMB, particularly OLDHs, but neither LDHs nor OLDHs can lower the service temperature limit of SMB.

## 4. Conclusions

LDHs organically modified by triethoxyvinylsilane were prepared and utilized to intensify their storage stability, dispersion, and anti-aging performance in SMB. Based on the experimental results, the major findings are as follows:

The storage stability of SMB and LSMB was poor, which was due to the floating upward of SBS and the segregation sinking of LDHs in bitumen. Compared with SMB and LSMB, OLSMB exhibited better storage stability. Triethoxyvinylsilane organic modification improved the dispersibility of LDHs in bitumen; in turn, the better dispersibility of OLDHs obstructed the movement of SBS in bitumen and enhanced the storage stability of SBS in bitumen.

The introduction of LDHs and OLDHs ameliorated the high-temperature behavior of SMB, and increased the decomposition temperature of the SBS network structure in bitumen, which could improve the thermostability of SBS in bitumen at a high temperature.

After aging, because of the aging of bitumen and the degradation of SBS, the rheological properties of SMB gravely deteriorated. The incorporation of LDHs and OLDHs can mitigate the aging damage on bitumen and SBS, reduce the deterioration of the rheological properties of SMB, and heighten the anti-aging ability of SMB. The effect of LDHs was ulteriorly strengthened after triethoxyvinylsilane organic modification.

SMB containing OLDHs exhibited excellent aging resistance, due to the better storage stability of OLDHs and SBS in bitumen, which indicated that the excellent storage stability is particularly important for SMB. Based on the result of this paper, it can be found that reducing the density difference between the modifier and SMB or inhibiting the movement of modifier in SMB can improve the storage stability of modifier in SMB. Hence, the follow-up research in these two aspects can be carried out to further improve the storage stability of SMB.

## Figures and Tables

**Table 1:** The basic properties of virgin bitumen and SBS.

Materials	Items	Properties
Bitumen	Penetration (25 °C, 0.1 mm)	73.0
	Ductility (10 °C, cm)	16.5
	Softening point (°C)	48.8
	Viscosity (135 °C, Pa·s)	0.49
SBS	Structure	Linear, 1301
	Block ratio (B/S)	70/30
	The average molecular weight (g/mol)	120,000

**Table 2:** Main parameters of DSR test.

Test Program	Test Temperature (°C)	D ^a^ (mm)	H ^b^ (mm)	Fre. ^c^ (rad/s)	Rat. ^d^ (°C/min)
Temperature sweep	−10–30	8	2	10	2
	30–80	25	1	10	2
Frequency sweep	−10, 0, 10, 20	8	2	0.01–400	-

^a^ Diameter of the plate, ^b^ gap between the plates, ^c^ frequency of sweep, ^d^ heating rate.

**Table 3:** The temperature of intersection points of all SMB samples.

Samples	The Temperature of Intersection Point (°C)		∆T (°C)
	Before Aging	After Aging	
SMB	15.8	27.4	11.6
LSMB	16.8	21.8	5.0
OLSMB	17.5	18.2	0.7

∆T = the temperature after aging−the temperature before aging.

## Data Availability

The raw/processed data required to reproduce these findings cannot be shared at this time as the data also form part of an ongoing study.
